# Molecular Characterization, Tissue Distribution and Expression, and Potential Antiviral Effects of TRIM32 in the Common Carp (*Cyprinus carpio*)

**DOI:** 10.3390/ijms17101693

**Published:** 2016-10-09

**Authors:** Yeda Wang, Zeming Li, Yuanan Lu, Guangfu Hu, Li Lin, Lingbing Zeng, Yong Zhou, Xueqin Liu

**Affiliations:** 1 College of Fisheries, Huazhong Agricultural University, Wuhan 430070, China; yedawang@webmail.hzau.edu.cn (Y.W.); lzmhhzzh@163.com (Z.L.); huguangfu@mail.hzau.edu.cn (G.H.); linli@mail.hzau.edu.cn (L.L.); 2 Freshwater Aquaculture Collaborative Innovation Center of Hubei Province, Wuhan 430070, China; 3 Key Laboratory of Freshwater Animal Breeding, Ministry of Agriculture, Wuhan 430070, China; 4 Department of Public Health Sciences, University of Hawaii at Manoa, Honolulu, HI 96822, USA; yuanan@hawaii.edu; 5 Yangtze River Fisheries Research Institute, Chinese Academy of Fishery Sciences, Wuhan 430070, China; zlb@yfi.ac.cn (L.Z.); zhouy@yfi.ac.cn (Y.Z.)

**Keywords:** TRIM32, spring viraemia of carp virus, common carp

## Abstract

Tripartite motif-containing protein 32 (TRIM32) belongs to the tripartite motif (TRIM) family, which consists of a large number of proteins containing a RING (Really Interesting New Gene) domain, one or two B-box domains, and coiled coil motif followed by different C-terminal domains. The TRIM family is known to be implicated in multiple cellular functions, including antiviral activity. However, it is presently unknown whether TRIM32 of common carp (*Cyprinus carpio*) has the antiviral effect. In this study, the sequence, expression, and antiviral function of TRIM32 homolog from common carp were analyzed. The full-length coding sequence region of *trim32* was cloned from common carp. The results showed that the expression of TRIM32 (mRNA) was highest in the brain, remained stably expressed during embryonic development, and significantly increased following spring viraemia of carp virus (SVCV) infection. Transient overexpression of TRIM32 in affected Epithelioma papulosum cyprinid cells led to significant decrease of SVCV production as compared to the control group. These results suggested a potentially important role of common carp TRIM32 in enhancing host immune response during SVCV infection both in vivo and in vitro.

## 1. Introduction

The tripartite motif (TRIM) family contains an N-terminal E3 ubiquitin ligase RING domain followed by one or two zinc-binding motifs named B-box; a predicted coiled coil (CC) region; and a variable C-terminus, such as a PRY/SPRY domain, also known as the B30.2 domain, or the NHL (NCL-1/HT2A/Lin-41 repeat) domain [1]. The TRIM family plays important roles in development, tumor suppression, disease pathology and viral restriction in mammals [2]. For example, TRIM5a was documented to have a restrictive ability against HIV-1 and other retroviruses [3,4]. TRIM13 has recently been reported to be a negative regulator of MDA5-mediated type I interferon production [5]. TRIM38 enabled to mediate lysosome-dependent degradation of TAB2/3 to inhibit the NF-κB signaling pathway [6]. TRIM37 was incorporated into HIV-1 particles and its expression could severely diminish viral DNA synthesis [7]. Human TRIM32 was able to modulate the signal pathway of type I interferon and showed antiviral activity through targeting MITA/STING protein for K63-linked ubiquitination [8]. A recent study by Liu and coworkers has demonstrated that TRIM32 negatively regulates tumor suppressor P53 [9].

There are also some studies to characterize TRIM proteins in teleost fish. TRIM33 from zebrafish (*Danio rerio*) is an essential regulator for embryonic and adult hematopoiesis [10]. Through deep sequencing technology, 58 and 240 TRIM/TRIM-like sequences were identified in pufferfish (*Tetraodon nigrovviridis*) and zebrafish, respectively [2,11]. Current studies have shown that a large number of TRIM genes are conserved among teleosts and mammals. Van der Aa et al. identified some multigene subsets of TRIM genes, a unique feature of fish [11]. In addition, this group conducted a hierarchical clustering analysis and identified 16 finTRIM clusters, which were up- or downregulated following viral hemorrhagic septicemia virus (VHSV) infection [12]. A recent study has indicated that *Epinephelus coioides* TRIM39 (EcTRIM39) not only plays an important role in cell cycle progression, but also possibly inhibits fish virus replication by acting as a regulator of innate immune response against fish viruses [13].

Spring viraemia of carp virus (SVCV) is a member of the genus *Vesiculovirus* in *Rhabdoviridae* family [14], and a highly pathogenic virus that has often caused excessive losses of cultured common carp [15]. However, effective treatment for SVCV infection has presently been very limited and this might partially be due to currently poor understanding of the viral pathogenic mechanism; no vaccine against SVCV is currently available [16]. Therefore, improved understanding of host immune responses to viral infection may facilitate the discovery of novel targets to control SVCV infection and replication in carp and other fish species.

In this report, we describe the inhibitory role of TRIM32 in common carp against SVCV infection, and have particularly defined such a role in downregulating viral replication. These new findings suggest a possible important role of TRIM32 in host immune response against viral infection in fish.

## 2. Result

### 2.1. Analysis of Common Carp trim32 Coding DNA Sequence (CDS) Region

**Figure 1:**
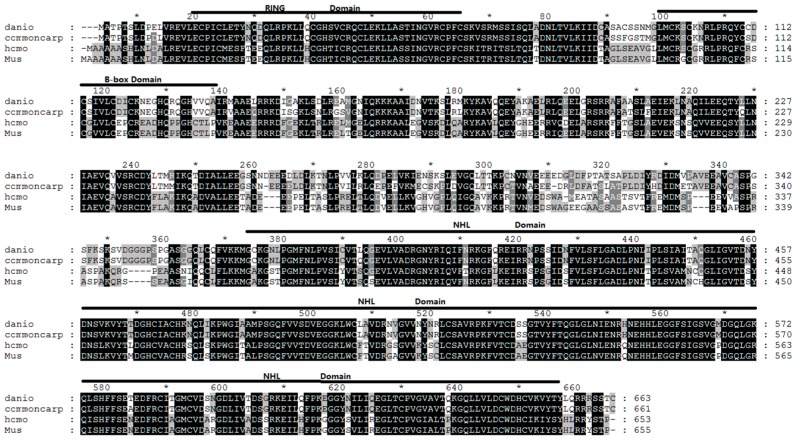
Multiple alignments of tripartite motif-containing protein 32 (TRIM32) amino acid sequences of *Danio rerio*, common carp, human, and mice. The amino acid sequence of common carp TRIM32 was predicted from the nucleotide sequence. The high conservation RING (AA^17^–AA^62^), B-box (AA^96^–AA^136^) and NHL (AA^369^–AA^651^) functional domain of TRIM32 across different species were marked with solid bold line; * means the middle number of the two numbers nearby it.

**Figure 2:**
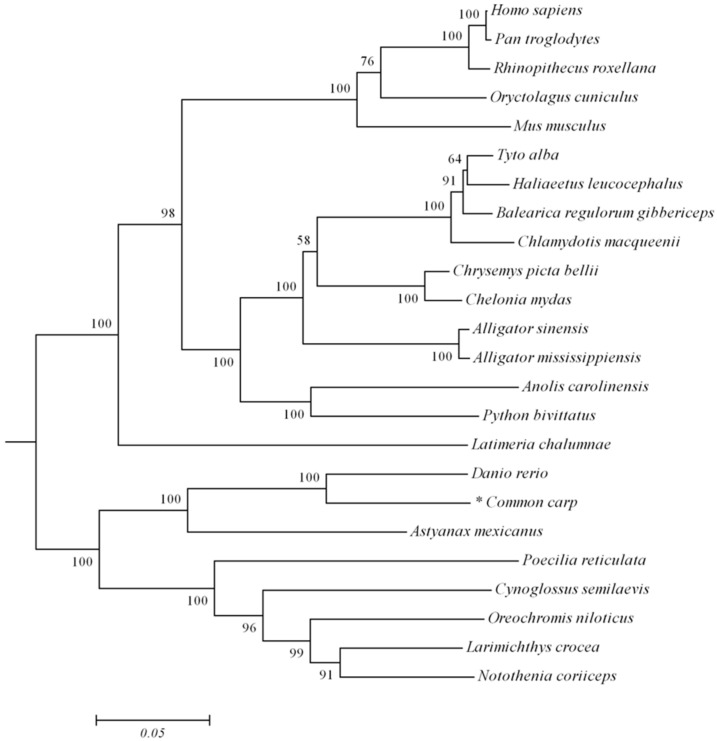
Phylogenetic analysis of TRIM32. Neighbor-joining tree was constructed with MEGA5.1, branches indicate confidence level of 10,000 bootstrap replications. All of the accession numbers used in this analysis are as follows: *Pan troglodytes*: XM_016961541.1; *Rhinopithecus roxellana*: XM_010381004.1; *Oryctolagus cuniculus*: XM_008273382.1; *Python bivittatus*: XM_007424916.2; *Anolis carolinensis*: XM_003227866.3; *Alligator mississippiensis*: XM_006267916.2; *Alligator sinensis*: XM_006026273.2; *Chelonia mydas*: XM_007057274.1; *Chrysemys picta bellii*: XM_008170837.1; *Chlamydotis macqueenii*: XM_010130338.1; *Balearica regulorum gibbericeps*: XM_010301630.1; *Haliaeetus leucocephalus*: XM_010571007.1; *Tyto alba*: XM_009972503.1; *Latimeria chalumnae*: XM_006011903.1; *Notothenia coriiceps*: XM_010779970.1; *Larimichthys crocea*: XM_010744032.1; *Oreochromis niloticus*: XM_005459485.1; *Cynoglossus semilaevis*: XM_008325928.2; *Poecilia reticulate*: XM_008424382.2; *Astyanax mexicanus*: XM_007252089.2; *Danio rerio*: NP_001107066.1; *Homo sapiens*: NP_001093149.1; and *Mus musculus*: EDL31081.1.

The full-length of *trim32* coding DNA sequence (CDS) region of the common carp was generated using RT-PCR and the 5’/3’-RACE technique. All the primers used in experimental tests are listed in Table 1. Sequence analysis showed that the *trim32* CDS region was 1986 bp in length and predicted a protein of 661 amino acid residues containing a highly conserved RING domain (AA^17^–AA^62^), a B-box domain (AA^96^–AA^136^), and an NHL domain (AA^369^–AA^651^). Multiple sequence alignments of these domains, which are marked with the solid bold line, indicated *trim32* is highly conserved among species with a few alternations at amino acid level (Figure 1). A phylogenetic tree generated with the software MEGA version 5.1 showed the evolutionary relationships of *trim32* from selected different species: common carp *trim32* and zebrafish *trim32* were clustered at one branch, while other species like mammal *trim32* were at another branch (Figure 2).

### 2.2. TRIM32 Tissue-Specific Distribution and Expression during Embryonic Development

**Figure 3:**
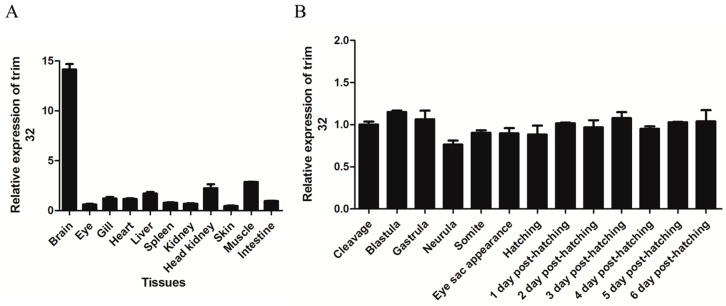
(**A**) The relative expression of common carp TRIM32 in different tissues obtained by qRT-PCR, the data was normalized to that in intestine; (**B**) Relative expression of TRIM32 during different stages of embryonic development, the data was normalized to that of the cleavage sample. TATA-box binding protein (TBP) was used as the internal control. Data presented here represent mean values from three independent experiments and error bars denote the standard deviation.

In order to characterize tissue-specific expression of TRIM32, 11 different tissues from three healthy common carps were collected and qRT-PCR was performed to quantify the mRNA level of TRIM32 in these tissues, with the use of TATA-box binding protein (TBP) as an internal control. Results showed that the expression of TRIM32 mRNA in liver, head kidney and muscle was high, especially the highest in the brain but the lowest in the skin. The quantity of TRIM32 in these tissues was normalized as compared to that in the intestine (Figure 3A). qRT-PCR was also used to detect the expression character of TRIM32 at the following stages of embryonic development: cleavage, blastula, gastrula, neurula, somite, eye sac appearance, hatching, and 6 days post-hatching. The results showed that the expression level of TRIM32 was stable with no significant change during embryonic development (Figure 3B).

### 2.3. Changes of TRIM32 Expression after SVCV Infection

**Figure 4:**
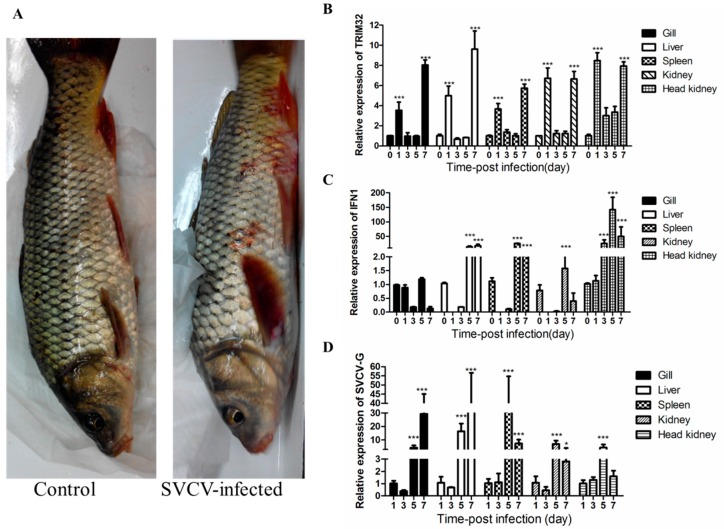
(**A**) Hemorrhagic symptoms appeared at day 5 post infection with SVCV compared with the fish in control group that did not show any symptom; (**B**–**D**) Relative expression patterns of common carp TRIM32, type 1 interferon (IFN1), and spring viraemia of carp virus glycoprotein G (SVCV-G) gene after SVCV infection, respectively. TBP was used as the internal control, the results were normalized basing on that of the carp at day 0 (for TRIM32 and IFN1) and the carp 1 day (for SVCV-G) after SVCV infection. Data presented here represent mean values from three independent experiments and error bars denote the standard deviation. The significant values (0.01 < *p* < 0.05 and *p* < 0.001) to the control group was noted with * and ***, respectively.

After SVCV infection, some test carps developed hemorrhagic symptoms (Figure 4A), relative changes of TRIM32 mRNA at different post-infection (p.i.) time was assessed using qRT-PCR. The results were normalized to that of the common carp at day 0 (infection with SVCV). As shown in Figure 4B, a very similar pattern of TRIM32 expression was observed in five tissues tested, showing a significant increase at p.i. day 1, then the expression decreased to approximately day 0 level at p.i. days 3 and 5, and then increased again and reached the highest level at p.i. day 7. Type 1 interferon (IFN1) and SVCV-G genes were also detected in gill, liver, spleen, kidney, and head kidney using qRT-PCR. As shown in Figure 4C,D, the relative mRNA level of IFN1 and SVCV glycoprotein G (SVCV-G) genes reached nearly the maximum at p.i. day 5, and this might suggest a relative complex relationship between them.

### 2.4. Expression of TRIM32 in Transfected EPC Cells

**Figure 5:**
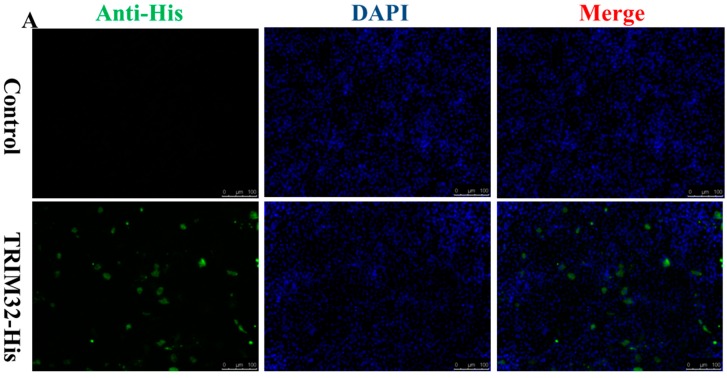

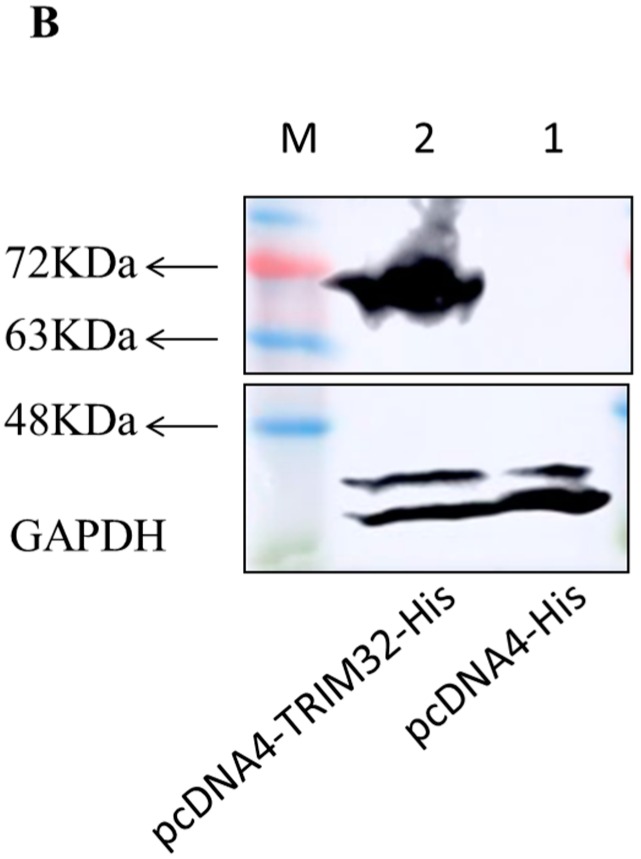
Immunofluorescence assay (IFA) and Western blotting assay to analyze the expression of TRIM32. (**A**) Immunofluorescence assay to detect the expression of recombinant plasmid pcDNA4-TRIM32-His in Epithelioma papulosum cyprinid (EPC) cell line, the empty vector was transfected as the control. Cell nucleus was stained by DAPI (4,6-diamidino-2-phenylindole); (**B**) Western blotting assay to detect the expression of recombinant plasmid pcDNA4–TRIM32–His in EPC cell line. **Lane 1** was transfected with pcDNA4–His empty vector as control while **Lane 2** was transfected with the recombinant plasmids pcDNA4–TRIM32–His, GAPDH (glyceraldehyde 3-phosphate dehydrogenase) was used as the internal control in this test.

Epithelioma papulosum cyprinid (EPC) cells were seeded in 6-well plates and transfected with the reconstructed plasmid pcDNA4–TRIM32–His. Sample cells were collected at 36 h after transfection for TRIM32 expression. As shown in Figure 5, high expression of TRIM32 in affected EPC cells were detected and verified by both immunofluorescence (IF) (Figure 5A) and Western blotting assays (Figure 5B).

### 2.5. Overexpression of TRIM32 Decreased the Virus Titer

**Figure 6:**
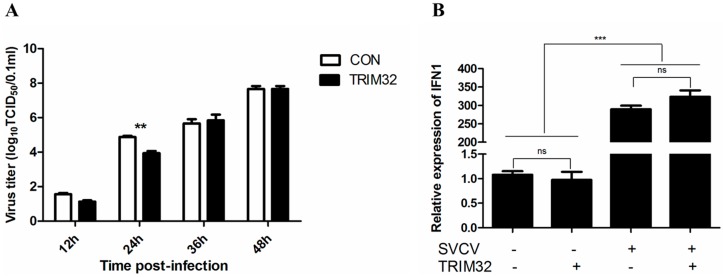
(**A**) Transient overexpression of TRIM32 in EPC cells may significantly decrease the virus titer after 24 h following SVCV infection. EPC cells with and without TRIM32 overexpression were infected with SVCV at 0.1 MOI, and virus titer (TCID_50_) was obtained from supernatant; (**B**) Overexpression of TRIM32 did not affect the mRNA level of IFN1 with or without SVCV stimulation, TBP was used as the internal control. Data presented here represent mean values from three independent experiments and error bars denote the standard deviation, the significant values (*p* < 0.01) to the control group was noted with ** and the significant values (*p* < 0.001) to the control group was noted with ***; ns means non-significant difference.

In order to verify the antiviral potential of TRIM32 in vitro, EPC cells with TRIM32 transient overexpression were infected with SVCV at a multiplicity of infection (MOI) of 0.1, and SVCV production was determined by TCID_50_ assay at four different p.i. time points. As shown in Figure 6, the overexpression of TRIM32 significantly decreased SVCV titer at 24 h post-infection (Figure 6A), but did not affect the mRNA level of IFN1 with or without SVCV stimulation (Figure 6B).

## 3. Discussion

In the present study, the full-length CDS region of common carp *trim32* was cloned and analyzed using bioinformatics technology. Sequence analysis revealed that the main functional domain of *trim32* of common carp shares high homology with many other species, including mammals. The domain is also homologous to that of zebrafish and matched with zebrafish in one cluster.

Analysis of tissue expression revealed that TRIM32 expression was highest in brain and immune-related tissues, such as the head, kidney, and liver. Other TRIMs including TRIM3a, were also known to express at high level in the brain of zebrafish and human, and human TRIM3, also referred to as brain-expressed RING finger protein (BERP), was not only originated from the brain but also associated with the central nervous system in humans as well as mice [17,18]. The TRIMs have been implicated in various antiviral effect [1,8] and our results were in agreement with these previous findings and suggested that common carp TRIM32 also played a role in carp fish defense against viral infection or in brain development.

Fertilized carp eggs were used to determine TRIM32 expression at various developmental stages and our results showed that the expression of TRIM32 was detected in all the experimental stages; the expression was stable throughout the early development. However, the mRNA level of TRIM32 in the SVCV-infected group was increased, especially at day 7 p.i. time as compared to that of the control group. This might suggest an important role of TRIM32 in antiviral activity or its potential effect on host inflammatory response.

To further characterize the function of TRIM32, a TRIM32 recombinant plasmid was constructed and well expressed in EPC cells. Transient overexpression of TRIM32 in transfected EPC cells showed a significant decline in viral production for a short time, SVCV titer then caught up with the longer incubation time. The mRNA level of IFN1 was not affected by the condition of TRIM32 overexpression and this suggested there might be some other signaling pathways involved in regulating the viral replication. The exact mechanism of this observation is currently not known, and future studies focusing on SVCV infection of cultures with constitutive or long-term expression of TRIM32 is needed to explain the observed effect. Previous studies showed that some TRIMs could inhibit viral entry into cells (e.g., TRIM11 and TRIM13), or interfere with later stages of viral activity including inhibiting viral release (e.g., TRIM25 and TRIM62) [1]. Further analyses are needed to determine the specific mechanism behind virus titer reduction by common carp TRIM32, such as testing for its RING E3 ligase activity [8] or interaction with endogenous protein [19,20].

TRIMs were previously shown to undergo antiviral activity and regulate inflammatory responses, such as human TRIM32-modulated type I interferon induction [8]. SVCV is a severe pathogen that continues to decimate cultured common carp populations and aquaculture industries worldwide. The present study provides an initial foundation arguing for future research critical to the discovery of innate immunity targets that may play a crucial role in SVCV prevention and control.

## 4. Materials and Methods

### 4.1. Cell and Virus

Epithelioma papulosum cyprinid (EPC, ATCC:CRL-2872) cell line was cultured at 28 °C in Eagle’s minimum essential medium (MEM, Hyclone, Logan, UT, USA) supplemented with 10% fetal bovine serum (FBS, Gibco, Melbourne, Australia), penicillin (100 µg/mL), and streptomycin (100 µg/mL). The spring viraemia of carp virus (SVCV, ATCC:VR-1390) was used in infection tests.

### 4.2. RNA Extraction and Synthesis of cDNA

Total RNA of common carp brain was extracted using TRIzol reagent (TaKaRa, Dalian, China) according to manufacturer’s protocol. One microgram of total RNA was used for the reverse transcription reaction using PrimeScript^TM^RT reagent Kit with a gDNA Eraser (TaKaRa); the cDNA was stored at −20 °C until used.

### 4.3. Cloning the Full-Length Coding DNA Sequence (CDS) of Common Carp trim32 Gene

To clone the full-length coding DNA sequence (CDS) of common carp *trim32*, RT-PCR and RACE techniques were used. Two pairs of degenerate primers corresponding to the highly conserved *trim32* sequences from zebrafish (*Danio rerio*), Tilapia (*Oreochromis* spp.), Swordfish (*Xiphias gladius*), and *Latimeria chalumnae* were designed to clone the core section of the *trim32* gene. Subsequently, the CDS region sequences were cloned through the RACE technique. 5’ RACE System for Rapid Amplification of cDNA Ends Version 2.0 Kit (Invitrogen, Shanghai, China) and SMARTer™ RACE cDNA Amplification Kit (Clontech, Palo Alto, CA, USA) were used to obtain the entire CDS region sequence of carp *trim32*. Next, specific primers were designed according to the sequence used to clone the full-length *trim32* gene, the gene accession number was KX388359.

### 4.4. Sequence Analysis

The trim32 gene sequence was confirmed by NCBI BLAST analyses (http://www.ncbi.nlm.nih.gov/blast) and its homology with other known sequences. All the accession numbers used in the analysis: *Danio rerio* TRIM32 (NP_001107066.1), *Homo sapiens* TRIM32 (NP_001093149.1), and *Mus musculus* TRIM32 (EDL31081.1). The CLUSTALW program was used for multiple sequence alignments, and the phylogenetic tree was constructed based on the amino acid sequences of TRIM32 using Neighbor-Joining algorithm using MEGA version 5.1 (http://softadvice.informer.com/Mega_5.1_Free_Download.html).

### 4.5. Experiment Common Carp and Common Carp Eggs

Common carp with an average mass of 150 g were purchased from Baishazhou fisheries (Wuhan, China) and kept in laboratory at water temperatures of 15–17 °C for seven days before experiments. Different developmental stages of common carp eggs such as cleavage, blastula, gastrula, neurula, somite, eye sac appearance, hatching and 6 days post hatching were collected from Yangtze River Fisheries Research Institute, Chinese Academy of Fishery Science, and stored at −80 °C for RNA extraction. All animal procedures were carried out strictly accordance with the recommendations in the Guide for the Care and Use of Laboratory Animals of the National Institutes of Health. All animal infections did not involve endangered or protected species and all of the experiments using common carps were performed under the approval of the Animal Ethics Committee of Huazhong Agriculture University (HZAU). The infection and dissection experiments were performed under 3-Aminobenzoic acid ethyl ester methanesulfonate (MS-222) anesthesia to minimize fish suffering.

### 4.6. Quantitative PCR Analysis of Tissue-Specific Distribution and Expression during Embryonic Development

The brain, eyes, gill, heart, liver, spleen, kidney, head kidney, intestine, skin, and muscle tissues were collected from three healthy carps and stored at −80 °C with TRIzol reagent for RNA isolation. Carp eggs were collected at different stages of embryonic development to characterize TRIM32 expression character. Primers for quantitative PCR were designed with Primer 5.0 based on target sequences. Real-time PCR was performed in a Roche machine using the SYBR Green qPCR SuperMix (TaKaRa) following the manufacturers’ instructions, and TBP (TATA box-binding protein) was used as the internal control. Each of experimental tests was repeated three times independently under the same conditions and the experimental results were presented as the mean ± S.D. (standard deviation) (*n* = 3).

### 4.7. Viral Infection

SVCV was propagated in the EPC cell line. Virus titer, given as tissue culture infection dose (TCID_50_/mL), was calculated by the method of Reed and Muench [21]. Thirty common carps with an average mass of 150 g were maintained in 200 gal tank of clean water at 16 °C, the optimal temperature for SVCV infectivity [15]. All of these carps were injected in the base of the pectoral fin with 30,000 TCID_50_ units of SVCV respectively at the same time. The head kidney, liver, gill, kidney and spleen tissues were isolated at 0, 1, 3, 5, and 7 days after injection and 3 carps were sampled for RNA extraction at each time point.

### 4.8. Plasmid Construction and Transfection

In order to understand the biological function of TRIM32, we constructed the recombinant eukaryotic expression plasmids pcDNA4–TRIM32–His by ligating the coding region of TRIM32 into pcDNA4 vector, which was digested with *Bam*HI and *Xho*I restriction enzymes. There is a His-tag on the downstream of the TRIM32 protein. The recombinant plasmid pcDNA4-TRIM32-His was transfected into EPC cells using Lipofectamine 2000 (Invitrogen, Shanghai, China) when the cells were approximately 60%–70% confluent according to the manufacturer’s protocol, cells transfected with empty vector pcDNA4-His as control.

### 4.9. Immune Fluorescence and Western Blotting Assay

Twenty-four hours after transfection, cells were washed with PBS, and then fixed with methanol for 10 min at room temperature and incubated with mouse anti-His-tag monoclonal antibody (1:300) for 1.5 h, and FITC (fluorescein isothiocyanate)-conjugated goat anti-mouse antibodies (ABclonal, Wuhan, China) for 1 h. These cells were stained with 4,6-diamidino-2-phenylindole (DAPI), cells were observed under fluorescence microscopy. For Western blot assay, cells were handled with RIPA lysis buffer (Beyotime, Shanghai, China) and subjected to SDS-PAGE, and transferred onto polyvinylidene fluoride membrane (PVDF) (Bio-Rad, Hercules, CA, USA). After blocking in 5% skim milk at room temperature for 30 min, the membrane was incubated with anti-His-tag monoclonal antibody (1:2000) from mouse for 2 h and washed 3 times with TBST (Tris Buffered Saline added Tween), Horseradish peroxidase (HRP) conjugated anti-mouse antibodies (ABclonal) from goat were subsequently used to incubate for 30 min, following another 3-time washes with TBST, the signal was detected with chemiluminescenct substrate (General Electric, Fairfield City, CT, USA).

### 4.10. Virus Titration

Twelve hours after transfection, EPC cells were infected with SVCV at a MOI of 0.1 and virus titer in supernatant was tested using TCID_50_ assay. EPC cells at their exponential phase were harvested and individual cell suspension was seeded in 96-well plates and cultured for 24 h. A SVCV stock was serially diluted 10-fold in MEM and 100 µL of diluted virus solution was added to each well. Affected cultures were incubated at 28 °C for more than 72 h and SVCV titer was calculated using the Reed–Muench method.

## 5. Conclusions

In this study we cloned the whole length CDS region of common carp TRIM32, and the characterization and expression profiles were described, the sequence was highly conserved among species, the expression of TRIM32 showed stable characterization during its early development stages, SVCV infection could stimulate the mRNA level of TRIM32. TRIM32 overexpression could significantly decrease the virus titer after 24 h following SVCV infection, this phenomenon was not related to the IFN1 signaling pathway.

## Figures and Tables

**Table 1:** Primers used in this study.

Primer	Sequence (5’ to 3’)	Application
Core segment Forward	TCCCAGCTGGCTGACaayytnacngt	PCR
Core segment Reverse	AGGCTTGATCAGCTGGTTCTtrtgrcangcna	PCR
TRIM32ORF Forward	AAAGGATCCATGGCCACACCAACATCTTTAG	Plasmid construction
TRIM32ORF Reverse	AAACTCGAGACATGTAGAGGAACGTCTCCTT	Plasmid construction
TRIM32 Forward	GGGTGGCAAGGGAAGT	qPCR
TRIM32 Reverse	GAGGAAGGGAGCAACAAT	qPCR
TBP Forward	TTACCCACCAGCAGTTTAG	qPCR
TBP Reverse	ACCTTGGCACCTGTGAGTA	qPCR
SVCV-G Forward	CGACCTGGATTAGACTTG	qPCR
SVCV-G Reverse	AATGTTCCGTTTCTCACT	qPCR
IFN1-Forward	GGTGAAGTTTCTTGCCCTGACCTTAG	qPCR
IFN1-Reverse	CCTTATGTGATGGCTGGTATCGGG	qPCR

## References

[1] Uchil P.D., Quinlan B.D., Chan W.T., Luna J.M., Mothes W. (2008). TRIM E3 ligases interfere with early and late stages of the retroviral life cycle. PLoS Pathog..

[2] Boudinot P., van der Aa L.M., Jouneau L., Du Pasquier L., Pontarotti P., Briolat V., Benmansour A., Levraud J.P. (2011). Origin and evolution of TRIM proteins: New insights from the complete TRIM repertoire of zebrafish and pufferfish. PLoS ONE.

[3] Nisole S., Stoye J.P., Saïb A. (2005). TRIM family proteins: Retroviral restriction and antiviral defence. Nat. Rev. Microbiol..

[4] Matthew S., Christopher M.O., Michel J.P., Michael K., Patrick A., Joseph S. (2004). The cytoplasmic body component TRIM5a restricts HIV-1 infection in Old World monkeys. Lett. Nat..

[5] Narayan K., Waggoner L., Pham S.T., Hendricks G.L., Waggoner S.N., Conlon J., Wang J.P., Fitzgerald K.A., Kang J. (2014). TRIM13 is a negative regulator of MDA5-mediated type I interferon production. J. Virol..

[6] Hu M.M., Yang Q., Zhang J., Liu S.M., Zhang Y., Lin H., Huang Z.F., Wang Y.Y., Zhang X.D., Zhong B. (2014). TRIM38 inhibits TNFα- and IL-1β-triggered NF-κB activation by mediating lysosome-dependent degradation of TAB2/3. Proc. Natl. Acad. Sci. USA.

[7] Tabah A.A., Tardif K., Mansky L.M. (2014). Anti-HIV-1 activity of Trim 37. J. Gen. Virol..

[8] Zhang J., Hu M.M., Wang Y.Y., Shu H.B. (2012). TRIM32 protein modulates type I interferon induction and cellular antiviral response by targeting MITA/STING protein for K63-linked ubiquitination. J. Biol. Chem..

[9] Liu J., Zhu Y., Hu W., Feng Z. (2014). TRIM32 is a novel negative regulator of p53. Mol. Cell. Oncol..

[10] Ransom D.G., Bahary N., Niss K., Traver D., Burns C., Trede N.S., Paffett-Lugassy N., Saganic W.J., Lim C.A., Hersey C. (2004). The zebrafish moonshine gene encodes transcriptional intermediary factor 1γ, an essential regulator of hematopoiesis. PLoS Biol..

[11] Van der Aa L.M., Levraud J.P., Yahmi M., Lauret E., Briolat V., Herbomel P., Benmansour A., Boudinot P. (2009). A large new subset of TRIM genes highly diversified by duplication and positive selection in teleost fish. BMC Biol..

[12] Van der Aa L.M., Jouneau L., Laplantine E., Bouchez O., van Kemenade L., Boudinot P. (2012). FinTRIMs, fish virus-inducible proteins with E3 ubiquitin ligase activity. Dev. Comp. Immunol..

[13] Wang W., Huang Y., Yu Y., Yang Y., Xu M., Chen X., Ni S., Qin Q., Huang X. (2016). Fish TRIM39 regulates cell cycle progression and exerts its antiviral function against iridovirus and nodavirus. Fish Shellfish Immunol..

[14] Liu Z., Teng Y., Xie X., Li H., Lv J., Gao L., Tian F., Jiang Y., Chu Z., Xie C. (2008). Development and evaluation of a one-step loop-mediated isothermal amplification for detection of spring viraemia of carp virus. J. Appl. Microbiol..

[15] Ahne W., Bjorklund H.V., Essbauer S., Fijan N., Kurath G., Winton J.R. (2002). Spring viremia of carp (SVC). Anat. Notes.

[16] Liu L., Zhu B., Wu S., Lin L., Liu G., Zhou Y., Wang W., Asim M., Yuan J., Li L. (2015). Spring viraemia of carp virus induces autophagy for necessary viral replication. Cell. Microbiol..

[17] Zhang X., Zhao H., Chen Y., Liu C., Meng K., Yang P., Wang Y., Wang G., Yao B. (2012). Characterization and biological function analysis of the *trim3a* gene from zebrafish (*Danio rerio*). Fish Shellfish Immunol..

[18] Boulay J.L., Stiefel U., Taylor E., Dolder B., Merlo A., Hirth F. (2009). Loss of heterozygosity of TRIM3 in malignant gliomas. BMC Cancer.

[19] Fu B., Wang L., Ding H., Schwamborn J.C., Li S., Dorf M.E. (2015). TRIM32 Senses and Restricts Influenza A Virus by Ubiquitination of PB1 Polymerase. PLoS Pathog..

[20] Perumal T.M., Gonzalez-Cano L., Hillje A.-L., Taher L., Makalowski W., Suzuki Y., Fuellen G., del Sol A., Schwamborn J.C. (2015). TRIM32 modulates pluripotency entry and exit by directly regulating Oct4 stability. Sci. Rep..

[21] Reed L., Muench H. (1938). Measurement of viruses by end-point dilution assay. Am. J. Hyg..

